# Evidence-Based Strategies for Mitigating Pancreatic Fistula After Distal Pancreatectomy: A Systematic Review of Randomized Clinical Trials

**DOI:** 10.3390/jcm15041433

**Published:** 2026-02-12

**Authors:** Gabriela Del Angel-Millán, Celeste del Basso, Fabio Giannone, Marco Palucci, Federico Sangiuolo, Igor Monsellato, Gianluca Cassese, Fabrizio Panaro

**Affiliations:** 1 Division of Hepato-Pancreato-Biliary, Oncologic and Robotic Surgery, Azienda Ospedaliero-Universitaria SS. Antonio e Biagio e Cesare Arrigo, Via Venezia 16, 15121 Alessandria, Italy; delangelmillang@gmail.com (G.D.A.-M.); marco.palucci@ospedale.al.it (M.P.);; 2 Department of Research and Innovation (DAIRI), Azienda Ospedaliero-Universitaria SS. Antonio e Biagio e Cesare Arrigo, Via Venezia 16, 15121 Alessandria, Italy; 3 Department of Health Sciences, School of Medicine, University of Eastern Piedmont “Amedeo Avogadro”, 15121 Alessandria, Italy

**Keywords:** distal pancreatectomy, postoperative pancreatic fistula, mitigation of pancreatic fistula, pancreatic closure technique

## Abstract

**Background:** Postoperative pancreatic fistula remains a frequent complication after distal pancreatectomy and represents the first cause for major morbidity and mortality. Multiple strategies have been proposed to mitigate the severity of pancreatic fistula, but their real benefits remain inconclusive. This study aimed to identify effective mitigation strategies for clinically relevant pancreatic fistula (CR-POPF) through a systematic review of randomized clinical trials. **Methods:** A systematic search of the Medline and Web of Science databases was conducted for studies published between 2006 and February 2025. Eligible studies included randomized clinical trials evaluating strategies to mitigate clinically relevant postoperative pancreatic fistula following distal pancreatectomy. Only studies in English and involving human subjects were included. **Results:** Twenty-seven studies were found eligible, comprising 4062 patients, treated with 22 different strategies classified in 8 categories: tissue coverage, sealants and glues, systemic corticoids, analogues of somatostatin, anastomosis of the stump, drain usage, closure of the stump and transpapillary stent. Only 6 studies demonstrated a significant reduction in CR-POPF, strategies applied include systemic corticoids, selective use of drains, polyglycolic acid mesh, reinforced staplers, and collagen enhanced thrombin sealant. **Conclusions:** Studies reporting successful strategies show considerable heterogeneity in both the included populations and the way the strategies were applied. A personalized approach based on the risk of developing fistula and specific pancreatic features may be beneficial and should be further explored in future randomized clinical trials.

## 1. Introduction

Among pancreatic surgeries, distal pancreatectomy (DP) has often been viewed as a less critical procedure, being technically less demanding and performed in a wider variety of clinical settings when compared to other pancreatic resections [1]. DP is the treatment of choice for benign and malignant tumors arising in the body and tail of the pancreas, such as pancreatic adenocarcinoma, intraductal papillary mucinous neoplasms with malignant transformation, and other large low-malignant and benign tumors. Other procedures may be considered for selected tumors, such as parenchyma-preserving pancreatic resections, usually decided upon multidisciplinary team evaluation; however, DP remains the most frequently performed procedure [2]. Despite multiple advances in perioperative management and surgical technology, postoperative pancreatic fistula (POPF) remains a common complication following DP, with reported rates ranging from 5 to 44% in various series [3,4]. According to the International Study Group for Pancreatic Surgery (ISGPS), POPF is defined as an output in the drain, surgically placed or subsequently inserted, containing amylase levels greater than three times the upper normal serum value, after postoperative day 3 [5]. The updated definition further distinguishes clinically relevant POPF (CR-POPF), which is associated with a deviation from the expected postoperative course, from a biochemical leak that lacks clinical impact, grades considered as clinically relevant are B and C from the initial definition [6].

CR-POPF may lead to secondary complications, including intra-abdominal abscess formation and sepsis, and remains the leading cause of major morbidity and mortality after distal pancreatectomy [7]. These complications can result in prolonged length of hospital stay, delayed recovery and increased healthcare costs [5]. For this, mitigating the incidence and severity of POPF has been a continued effort for pancreatic surgeons. Numerous strategies have been proposed to achieve this goal in distal pancreatectomy, including variations in stump closure techniques, creation of a pancreatic anastomosis, application of glues or mesh reinforcement, routine drain placement, and systemic treatments with corticosteroids or somatostatin analogues. However, the evidence supporting these strategies remains inconclusive, and no consensus has yet been established [2,4,7,8,9,10,11,12,13,14,15,16,17,18,19,20,21,22,23,24,25,26,27,28].

The aim of this study is to provide a comprehensive summary of high-quality evidence assessing the effectiveness of these strategies for mitigating CR-POPF following a DP.

## 2. Methods

### 2.1. Search Strategy

A search was performed in two electronic databases, Medline (Pubmed) and Web of Science; using the terms (“left pancreatectomy”) OR (“distal pancreatectomy”) OR (“partial pancreatectomy”) OR (“pancreatectomy”) OR (“pancreato splenectomy”) OR (“spleno pancreatectomy”) AND (“pancreatic fistula”) OR (“postoperative complications) OR (“stump closure”) OR (“handsewn”) OR (“surgical stapler”) OR (“pancreatic anastomosis”) OR (“pancreaticojejunostomy”) OR (“pancreaticogastrostomy”) OR (“electrocoagulation”) OR (“tissue link”) OR (“ultrasonic activated device”) OR (“fibrin sealant”) OR (“sphincter of Oddi”) OR (“stents”) OR (“patch”) OR (“coverage”) OR (“drainage”) OR (“drain”) OR (“somatostatin”) OR (“octreotide”) OR (“lanreotide”) OR (“pasireotide”) OR (“laparoscopy”) OR (“laparoscopic surgery”) OR (“robotic surgery”) OR (“minimally invasive surgery”) OR (“seamguard”) OR (“Tachosil”) OR (“Noveil”) OR (“corticoids”) OR (“prednisone”), to retrieve randomized clinical trials (RCT) published from January 2006 until February 2025. The search period was established to ensure the use of the first ISGPS definition and grading of POPF in A, B, and C, defining CR-POPF as those classified as B and C [5,6]. An additional search was conducted through the references of selected articles to identify other suitable studies. Search methods followed the PRISMA (Preferred Reporting Items for Systematic Reviews and Meta-Analysis) guidelines [29]. Two authors (GDAM, CDB) independently reviewed the search results and selected potential articles based on title and abstract for inclusion, subsequently, they performed full-text revision and data extraction. In case of disagreement regarding inclusion, a third reviewer was involved (FG). This Systematic Review is registered in PROSPERO with an ID: 1248791.

#### Study Selection

The criteria for included articles were as follows: (i) prospective RCT investigating strategies for mitigating POPF in DP, (ii) studies using the ISGPS definition for POPF and classification, (iii) full published and (iv) English written articles. The exclusion criteria were: (i) articles not reporting rates of CR-POPF (grades B/C) specifically for DP, (ii) publications without an available full text or in (iii) languages other than English, (iv) video and conference abstracts, (v) other study types (meta-analysis, review articles, animal studies or duplicate studies, prospective and retrospective non-randomized studies, trial protocols, systematic reviews, meta-analyses), (vi) secondary publications of previously published studies.

### 2.2. Data Extraction

Data were retrieved by two researchers (GDAM, CDB). Extracted data included the first author’s name, publication year, title, journal, country, article type, number of patients, strategies used to mitigate POPF, intraoperative data (type of approach for DP, pancreas texture, diameter of pancreatic duct, use of drain), postoperative outcomes (CR-POPF- grades B and C, total POPF- grades A, B and C), abdominal collection, length of hospital stay, major complications according to the Clavien-Dindo System, postoperative mortality at 30 an 90 days), as well as costs and adverse events associated with each strategy [6,30].

The primary outcome was the rate of CR-POPF after each strategy. All other postoperative events were assessed as secondary outcomes.

### 2.3. Statistical Analysis

Data was summarized using descriptive statistics. Continuous variables are presented in mean and standard deviation, as well as median and range or interquartile range depending on each study, categorical variables are summarized in incidences and percentages. Weighted median and mean were calculated when no value between the groups was available in the same study. These weighted values were calculated only for descriptive purposes, using the study sample size as the weighting factor, and were not intended for inferential or comparative analysis. They were included solely to illustrate general trends across studies. Statistical analyses were performed using IBM SPSS Statistics, version 23.0 (IBM Corp., Armonk, NY, USA).

### 2.4. Bias Assessment and Certainty of the Evidence

The quality of included studies was assessed by two researchers (GDAM, CDB) using the Cochrane’s ROB 2 Assessment Tool. This tool evaluates bias across five domains: randomization process, deviations form intended interventions, missing data, measurement of outcomes and selection of reported results [31]. In addition, we evaluated the certainty of the evidence for each category of mitigation strategies using the GRADE (Grading of Recommendations Assessment, Development and Evaluation) approach. This included the evaluation of five domains: risk of bias, inconsistency, indirectness, imprecision, and publication bias. For each strategy category, the overall certainty of the evidence was rated as high, moderate, low, or very low [32,33].

## 3. Results

### 3.1. Studies’ Characteristics

**Figure 1:**
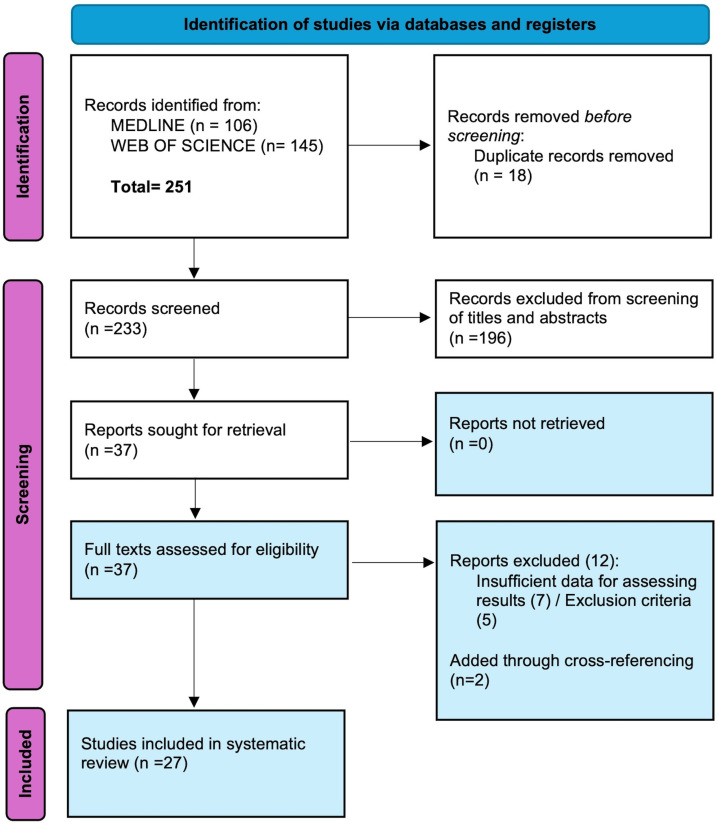
PRISMA Flowchart illustrating search methods and selection of the studies.

The literature search identified 251 RCTs, 37 studies were selected for full-text revision. Seven articles were excluded for not evaluating outcomes specific to DP, and five studies did not assess proper mitigation strategies, resulting in 25 studies. Two additional studies were retrieved through cross-referencing. Ultimately, 27 studies were included in the final review. Figure 1 [3,7,8,11,12,13,14,15,16,18,19,22,23,24,25,26,27,28,34,35,36,37,38,39,40,41,42].

**Figure 2:**
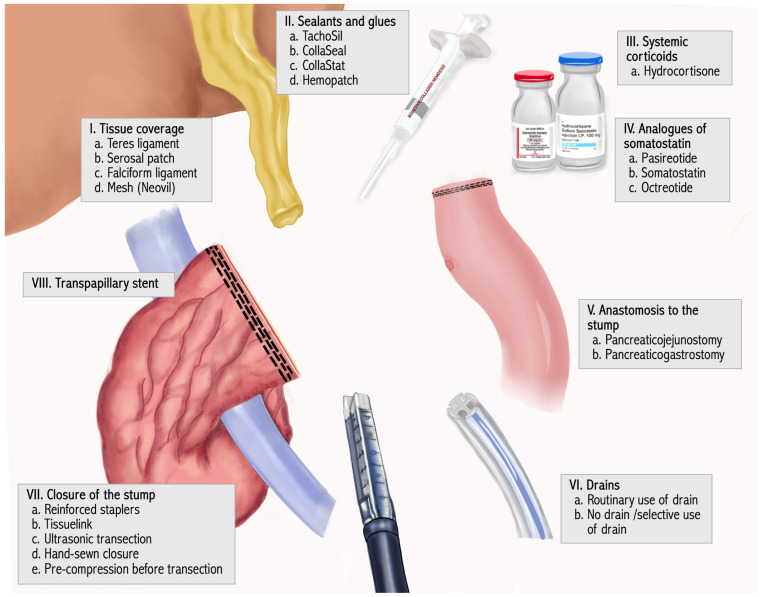
Categories of the specific strategies. Eight categories including 22 strategies.

The RCTs included a total of 4062 patients treated with 22 different mitigation strategies, which were classified in 8 categories. The cluster strategy is shown in Figure 2.

Details of the included studies are summarized in Table 1. Eleven studies stratified the randomization process based on key factors, including participating institution, annual center volume, type of surgery, neoadjuvant therapy, pancreatic texture, dilatation of the pancreatic duct and fistula risk [7,12,18,19,22,23,26,28,39,41,42]. An open approach for DP was the most used, performed in 2325 (57.2%) patients, while 1171 (28.8%) underwent a laparoscopic DP, 148 (3.6%) a robotic DP, and the approach for 412 (10.1%) patients was not reported. Pancreatic texture was reported by 14 studies, a soft/normal pancreas was found in 1981 (87.1%) patients, while firm or hard pancreas was reported in 287 (12.6%) [7,12,13,14,15,18,19,22,27,28,34,35,37,39,41]. Four studies conducted subgroup analysis based on pancreatic texture. Pancreatic duct diameter was reported by 11 studies, typically as mean (or median) values, 4 studies used a binary cutoff of 3 mm, while one RCT included only patients with a duct < 4 mm in diameter [7,8,11,13,14,19,22,23,27,39,41]. Pancreatic thickness at the transection line was reported in 6 studies, encompassing 1286 patients [7,11,18,23,25,27,28]. Three studies performed a stratified analysis based on pancreas thickness [18,28,41].

### 3.2. Clinically Relevant Postoperative Pancreatic Fistula

The cumulative incidence rate of CR-POPF was 20.1%, occurring in 816 patients, regardless the strategy applied. Six studies reported a significant reduction in the rate of CR-POPF, use of a polyglycolic mesh (Neovil), a collagen and thrombin sealant (Collastat), perioperative hydrocortisone, selective use of drainage, reinforced staplers and handsewn closure, each strategy belonging from a different category group and supported only by one study [13,14,23,27,36,38]. Among the four strategies assessing tissue coverage of the stump, only the use of Neovil significantly reduced the rate of CR-POPF compared to a non-intervened group (*n* = 5 (11.4%) vs. *n* = 15 (28.3%), respectively, *p* = 0.04) [27]. In the sealants and glues category, TachoSil failed to demonstrate any benefit in several studies, whereas Collastat proved superiority over a collagen sealant enhanced with fibrin (Collaseal), [*n* = 6 (46.2%) vs. *n* = 1 (8.3%), respectively, *p* = 0.027] [13]. Perioperative hydrocortisone use was also found beneficial compared to placebo in intended high-risk patients (*p* = 0.028), but not when compared with a somatostatin analogue [14,43]. The PANDORINA trial succeed in proving a significant reduction in CR-POPF using a selective drain strategy instead of the routine use of drains [*n* = 16 (12%) vs. *n* = 39 (27%), *p* < 0.001] [23]. Regarding stump closure techniques, Hamilton et al. was the only study among those included significantly reducing rate of CR-POPF using reinforced staplers against conventional ones (*p* < 0.007) [38]. Pancreatic anastomosis did not confer any benefit and was even detrimental compared to hand-sewn closure in some cases [19,28,36]. The use of analogues of somatostatin and the use of a transpapillary stents failed in mitigating CR-POPF. Incidences of CR-POPF for each strategy is displayed in Table 1. Subgroup analyses based on pancreas or patient related features were reported in three studies. Kawai et al. observed a non-significant trend toward reduced CR-POPF with pancreatojejunostomy in patients with pancreatic thickness > 12 mm, (*p* = 0.08), when compared to conventional stapling closure [28]. Kondo et al., reported a benefit of reinforced staplers when pancreatic section line was smaller to 14 mm, (*p* = 0.01) [18]. Van Bodegraven et al., in a post-hoc analysis, proved that in patients with low risk of POPF, the no-drain strategy carried a risk reduction of 17.9% for developing a CR-POPF [23]. Sumiyoshi et al., reported lower CR-POPF rates when using a pre-compression/pre-firing stapling strategy in patients with BMI > 25 kg/m^2^ [41].

### 3.3. Secondary Postoperative Outcomes

Two specific strategies, which showed no benefit in the main outcome assessment, were, on the contrary, able to significantly reduce the overall rate of POPF (Jejunal serosal patch and hydrocortisone versus somatostatin analogues) [8,40]. Among the strategies effective for CR-POPF mitigation, only three were also associated with a reduced incidence of overall POPF (Handsewn versus pancreatic anastomosis, reinforced staplers and Collastat) [13,36,38]. Regarding other postoperative outcomes, the incidence of abdominal collections was significantly reduced using a pancreaticogastric anastomosis and routine drainage [19,22]. In contrast, Landoni et al. found a higher rate of collections in the ultrasonic transection group compared to the conventional stapler group [25]. Major complication rate was higher in the intervention group in four studies [8,23,25,34]. Mungroop et al., reported a shorter length of hospital stay in 25 patients who used TachoSil over the pancreatic stump, while mortality rate was not influenced by any specific intervention [11]. Secondary outcomes are shown in Table 2.

Costs related to the strategies were not consistently reported. Only two trials provided cost information: Antila et al. reported lower treatment costs for hydrocortisone compared to pasireotide, and Uranues et al. found reduced total hospital costs associated with Hemopatch in patients who developed POPF [14,15].

No significant strategy-related adverse events were reported in the included RCTs. Sa Cunha et al. described 85 events possibly related to TachoSil application, though none led to postoperative complications [37]. Uemura et al., reported an average increase in operative time of 70 min when pancreaticogastrostomy was performed [19].

### 3.4. Bias Assessment and Certainty Analysis

As a result of the application of the ROB 2 tool, for evaluating potential bias in the studies, 12 studies were found with some concerns while the rest of them had a low risk for bias. Among the studies with some concerns, the domains most frequently affected were the randomization process and deviations from intended interventions, mainly due to limited reporting of allocation concealment and absence of blinding. Full assessment is shown in Table S1.

The overall certainty of the evidence for each category of mitigation strategy was assessed using the GRADE approach. Among the evaluated categories, only selective use of abdominal drains reached a moderate level of certainty. Two strategies, transpapillary stents and systemic administration of somatostatin analogues, were graded as very low certainty. The remaining strategies were classified as having low certainty. In the GRADE assessment, downgrading of certainty was primarily related to imprecision (small sample sizes, wide confidence intervals) and heterogeneity between trials, especially when evidence was derived from a single study. Full details of the GRADE assessment are presented in Table S2.

## 4. Discussion

CR-POPF remains the major determinant of morbidity and mortality following DP [44]. Although numerous strategies have been proposed to mitigate this complication, including the development of a fistula risk score, there is still no standardized approach for pancreatic transection or stump closure that effectively reduces the incidence of CR-POPF [2,44,45]. In summary, this systematic review identified multiple strategies, however, only six specific strategies were found to be successful in mitigating CR-POPF after DP. These positive results belong to different clusters, and they were not supported by other studies assessing the same intervention or comparable techniques within the same category. Additionally, two of the studies reporting a benefit, compared two active interventions, rather than using a conventional closure or placebo, which limits the generalizability of their findings. Moreover, despite the well-established link between CR-POPF and postoperative morbidity, these six strategies were rarely associated with improvements in related outcomes such as abdominal collections, major complications or length of hospital stay.

Synthetizing data from the included RCTs to determine effective mitigation strategies could be challenging for several reasons. For instance, reinforced staplers were suggested to be beneficial by Hamilton, et al., however, only one of the four studies assessing this specific strategy supported this finding. The use of a reinforced stapler is thought to provide extra support during the stump closure, despite this, current evidence from other systematic reviews does not support their effectiveness, and no specific type of reinforcement has been shown to be superior [38,45]. Other commonly explored approaches, such as pancreatic anastomosis or the use of pharmacological agents (oral or subcutaneous), proved largely ineffective based on the findings of this current review. Notably, Antila et al., reported a higher rate of CR-POPF following pancreaticojejunal anastomosis compared to handsewn closure [36]. The main indication for a handsewn technique is a thick pancreas, where a stapler may not be suitable. On the other hand, the use of an anastomosis is not widely performed, and the result may depend on the approach and technical skills, for this, results of this study cannot be generalized [46].

Another frequently used tool, the TachoSil, was evaluated in four studies and showed no significant impact on CR-POPF rates. These findings are corroborated by several meta-analyses, that similarly found no difference in CR-POPF incidence with the use of this sealant after DP [47,48]. Among sealants, Collastat a collagen and thrombin enhanced matrix was the only agent shown to be effective. Unlike Tachosil, Collastat not only enhances coagulation cascade through thrombin, but also promotes platelet aggregation. However, despite the positive results, the study was underpowered and remains the only study evaluating this type of sealant, therefore, its use cannot be standardized [49]. The only coverage technique potentially useful in this setting is Neovil, a polyglycolic acid mesh, however, only one study evaluating this strategy was included in our systematic review. The use of Neovil theoretically induces local inflammation which leads to the formation of granulation tissue sealing the surgical bed and potentially avoiding leak. Recently another study reported a combination of Neovil with handsewn closure reporting a lower rate of CR-POPF also advising its benefit in preventing traumatic laceration of the pancreatic tissue [50]. This was recently supported in a meta-analysis exploring this type of reinforcement, where a 26% risk reduction of CR-POPF was found when used in partial pancreatectomies [51].

In clinical practice, however, it is well recognized that surgical techniques alone do not determine the occurrence of POPF. Emerging evidence suggests that the efficacy of these interventions may depend on patient-specific baseline characteristics, supporting the need for a personalized rather than a universal approach. The non-stratified inclusion of low-risk patients in many of these studies may have biased the outcomes and diminished the apparent efficacy of certain strategies. The recent development and validation of a fistula risk score specific to DP (D-FRS) offers an opportunity to better stratify patients, enhance study design, and improve the methodological quality of future RCTs [52,53]. This concept was applied in the recent PANDORINA trial, where the no-drain policy in patients at low-risk patients reduced the rates of major morbidity and CR-POPF [23]. As a result of this, the strategy of selective use of drains was found successful in this review. It is worth to notice that despite this result, a recent Italian survey reported that fewer than 1% of surgeons apply this strategy, and less than 10% adjust their intraoperative approach based on risk assessment [54]. The rationale behind this strategy lies in existing evidence suggesting that routine drainage after pancreatectomy may be detrimental, mainly due to local irritation caused by the drain and the negative pressure typically applied in such systems [55].

Another example of a personalized approach was the success of the perioperative hydrocortisone, which was tested on a cohort of patients with soft pancreas only [14]. The use of perioperative steroids is based in the potential of these drugs to ameliorate the inflammatory response occurred during surgery and pancreatic manipulation [56]. Particularly in high-risk populations, such as patients with soft pancreas, there may be a benefit, as soft pancreas is more prone to fracture during transection and ischemia at the section margin [57].

Similar subgroup analyses in other RCTs echoed this personalized strategy; for instance, reinforced staplers were found to be effective in mitigating CR-POPF when used in patients with a thin pancreas or a BMI > 25 kg/m^2^ [18,58]. Also, a pre-compression strategy before firing is useful in this last group [41].

From a clinical standpoint, these findings emphasize the importance of adopting a personalized, risk-adapted approach to POPF prevention based on a standardized tool like the D-FRS.

Finally, it should be emphasized that comparing the assessed strategies is challenging, mainly due to variation in pancreatic features, patient selection criteria and changes in methods for implementing the strategy. Even the closure technique before application of the strategies applied differed widely among the studies, some using conventional stapler, hand-sewn techniques or other devices at the surgeon’s discretion. Moreover, when staplers are used routinely, additional factors such as cartridge type, compression time, and tissue thickness may significantly influence outcomes [59]. Indeed, a large retrospective study by Kang et al., showed that staplers with a 1.8 mm cartridge may be more suitable for thin pancreas in terms of bleeding, compression and the incidence of POPF. On the contrary, no significant benefit was observed with specific cartridge types in case of pancreases with greater thickness [60].

The strengths of this systematic review lie on the inclusion of RCTs with homogeneity in reporting the main outcome, providing high-quality evidence. Nonetheless, this review has several limitations, including patient heterogeneity, comparisons between active strategies rather than versus placebo or standard care in some trials, and variations in the implementation of each intervention, factors that make objective comparisons difficult. Moreover, the small number of RCTs among the strategy categories, combined with inconsistent use of risk stratification and personalized approaches, limits generalizability.

Although a formal meta-analysis and statistical assessment of publication bias would be desirable, the marked heterogeneity between the studies, precluded a meaningful quantitative synthesis. Performing such analyses under these conditions might have led to misleading conclusions. This limitation underscores the need for future standardized RCTs that will allow robust meta-analytical comparisons. This presents an opportunity for future RCTs to incorporate patient stratification based on fistula risk scores and to standardize methods for stump closure and postoperative management, enabling more precise conclusions.

## 5. Conclusions

There is no evidence supporting a universal mitigation strategy for all patients undergoing DP. However, certain risk-based approaches may help reduce the incidence of clinically relevant postoperative pancreatic fistula (CR-POPF). These include a no-drain policy for low-risk patients, perioperative hydrocortisone for high-risk patients, reinforced staplers and pre-compression for overweight patients, and the use of polyglycolic acid mesh. Larger randomized clinical trials evaluating the personalized implementation of these strategies are needed to draw definitive conclusions.

## Figures and Tables

**Table 1:** Characteristics of Included Studies, Patient Features, and Main Outcome.

Strategy Category	Strategy Applied vs. Control Group	Author, Publication Year	Multicentric Study (Yes/No)	Number of Patients (*n*)	Pancreatic Features of Included Patients			Incidence of CR-POPF		*p* Value
					Soft Pancreas *n* (%)	Wirsung Diameter (mm)/*n* (%)	Thickness of the Transection Line (mm)	Intervention Group *n* (%)	Control Group *n* (%)	
Tissue coverage	Jejunal serosal patch vs. stapler closure	Olah, 2009 [40]	No	70	NR	NR	NR	1 (2.87)	3 (8.57)	0.428
	Falciform patch + fibrin glue vs. standard closure *	Carter, 2013 [35]	Yes	101	101 (100)	NR	NR	9 (18)	9 (17.65)	1.000
	Teres ligament patch vs. no coverage	Hassenpflug, 2016 [16]	No	152	NR	NR	NR	17 (22.36)	25 (32.89)	0.146
	Polyglycolic acid mesh (Neoveil) vs. no intervention	Jang, 2017 [27]	Yes	97	84 (86.59)	<3 mm: 72 (74.23)	16.63 mm ^+^	5 (11.4)	15 (28.3)	***0.04***
Sealants and glues	Fibrinogen and Thrombin matrix (TachoSil) vs. no intervention	Montorsi, 2012 [39]	Yes	275	236 (85.82)	<3 mm: 204 (74.18)	NR	12 (8)	18 (14)	0.139
	Fibrinogen and Thrombin matrix (TachoSil) vs. no intervention	Sa Cunha, 2015 [37]	Yes	270	251 (92.96)	NR	NR	41 (30.6)	33 (24.3)	0.276
	Fibrinogen and Thrombin matrix (TachoSil) vs. no intervention	Park, 2016 [7]	Yes	101	85 (84.16)	<3 mm: 70 (60.31)	16.5 mm ^+^	11 (22.9)	15 (28.3)	0.536
	Fibrinogen and Thrombin matrix (TachoSil) vs. no intervention	Mungroop, 2021 [11]	No	247	NR	2 mm ^Δ^	13 mm ^Δ^	25 (20)	29 (24)	0.539
	Collagen enhanced fibrin sealant (CollaSeal) vs. Collagen enhanced thrombin sealant (CollaStat)	Park, 2020 [13]	No	25	NR	NR	NR	6 (46.2)	1 (8.3)	***0.027***
	Collagen enhanced with PEG (Hemopatch) vs. no intervention	Uranues, 2021 [15]	Yes	315	NR	NR	NR	26 (16.25)	36 (23.22)	0.590
Systemic corticoids	Perioperative hydrocortisone vs. placebo	Antila, 2019 [14]	No	31	31 (100)	<3 mm: 29 (93.55)	NR	1 (6)	6 (43)	***0.028***
	Hydrocortisone vs. pasireotide *	Tarvainen, 2020 [8]	No	60	NR	<4 mm: 60 (100)	NR	6 (20)	4 (13)	0.488
Analogues of somatostatin	Somatostatin vs. octreotide	Gaujoux, 2024 [26]	Yes	170	NR	NR	NR	15 (17.6)	14 (16.5)	0.84
Anastomosis of the pancreatic stump	Pancreatico-jejunosto-anastomosis vs. hand-sewn closure *	Antila 2014 [36]	No	13	NR	NR	NR	3 (60)	1 (12.5)	**<*0.05***
	Pancreatico-jejunosto-anastomosis vs. conventional stapler	Kawai, 2016 [28]	Yes	123	110 (89.43)	NR	13.21 mm ^+^	6 (10.3)	10 (13.1)	0.334
	Pancreatico-gastro-anastomosis vs. handsewn closure *	Uemura, 2017 [19]	Yes	73	49 (67.12)	2.2 mm ^+^	NR	7 (19.44)	7 (18.91)	1.000
Routine use of drains	Routine drainage vs. selective drainage	Van Bodegraven, 2024 [23]	Yes	282	NR	1 mm ^Δ^	12 mm ^Δ^	39 (27)	16 (12)	**<*0.0001***
	Routine drainage vs. no drainage	Van Buren, 2017 [22]	Yes	344	273 (81)	2 mm ^Δ^	NR	31 (18)	20 (12)	0.114
Closure of the stump	Reinforced staplers vs. conventional stapler	Hamilton, 2012 [38]	No	100	NR	NR	NR	1 (1.9)	11 (23.91)	***0.0007***
	Reinforced staplers vs. conventional stapler	Kondo, 2019 [18]	Yes	120	111 (92.5)	NR	12.25 mm ^+^	10 (16.3)	16 (27.1)	0.15
	Reinforced staplers vs. conventional stapler	Wennerblom, 2021 [24]	Yes	106	NR	NR	NR	6 (11)	8 (16)	0.332
	Reinforced staplers vs. conventional stapler	Merdrignac, 2022 [12]	Yes	199	162 (81.41)	NR	NR	14 (14)	11 (11.1)	0.54
	Reinforced staplers vs. Radiofrequency + saline transection	Shubert, 2016 [3]	Yes	67	NR	NR	NR	4 (12.5)	8 (22.9)	0.35
	Ultrasonic transection vs. conventional stapler	Landoni, 2022 [25]	Yes	145	NR	NR	12 mm ^Δ^	14 (19)	9 (12)	0.191
	Hand-sewn vs. conventional stapler	Diener, 2011 [42]	Yes	352	NR	NR	NR	36 (20.34)	36 (20.57)	0.92
	Pre-compression vs. No-compression before firing	Sumiyoshi, 2025 [41]	Yes	171	153 (89.47)	2 mm ^Δ^	11.49 mm ^Δ^	11 (12.5)	11 (13.3)	0.833
Transpapillary stent	Transpapillary stent vs. no intervention	Frozanpor, 2012 [34]	No	53	48 (90.57)	NR	NR	11 (42.3)	6 (22.2)	0.122

NR: Not reported; * The comparing groups involve strategies from 2 or more different sections; ^Δ^ Value reported in median; ^+^ Value reported in mean. The figures in bold and italics are to highlight significant values.

**Table 2:** Postoperative complications after distal pancreatectomy, reported in the included studies.

Strategy Category	Study	Total POPF Rate (A/B/C) *n* (%)		*p* Value	Abdominal Collection *n* (%)		*p* Value	Major Complications *n* (%)		*p* Value	Postoperative Mortality (90-Day)		*p* Value	Length of Hospital Stay Median (Range)		*p* Value
Tissue coverage of the pancreatic stump	Olah [40]	3 (8.57)	7 (20)	***0.041***	1 (2.86)	3 (8.57)	***0.041***	2 (5.71)	5 (14.29)	0.428	1 (2.85) *	0 (0) *	NR	10 (5–20)	9 (6–20)	0.10
	Carter [35]	10 (20)	10 (19.61)	1	NR			NR			0 (0) *	0 (0) *	1	5 (4–20)	5 (4–95)	0.621
	Hassenpflug [16]	36 (47.4)	39 (51.3)	0.626	32 (53.1)	37 (56.1)	0.749	NR			0 (0) *	1 (1.3) *	0.316	11.5 (7–128)	12 (6–88)	0.843
	Jang [27]	29 (65.9)	29 (54.7)	0.26	19 (43.18)	23 (43.39)	NR	NR			0 (0)	0 (0)	NR	NR		
Sealants and glues	Montorsi [39]	90 (62.01)	89 (68.46)	0.267	39 (26.9)	30 (22.22)	NR	NR			0(0)	0(0)	NR	10 (6–33)	10 (6–55)	0.273
	Sa Cunha [37]	73 (54.5)	77 (56.6)	0.807	NR			NR			1 (0.75)	2 (1.47)	NR	17.5 ± 13.8 ^+^	14.9 ± 8.2 ^+^	0.324
	Park [7]	34 (70.8)	29 (54.7)	0.095	26 (54.2)	23 (43.4)	0.279	6 (12.5)	4 (7.5)	0.405	0 (0)	0 (0)	0.999	9.7 ± 3.4 ^+^	10 ± 6.3 ^+^	0.279
	Mungroop [11]	NR			NR			30 (24)	36 (30)	0.389	2 (1.6)	6 (4.9)	0.168	7 (5–9)	8 (6–11)	***0.025***
	Park [13]	8 (30.8)	16 (59.3)	***0.037***	NR			3 (23.1)	1 (8.3)	0.593	0 (0)	0 (0)	0.999	7 (6–9) ^Δ^	7 (6–14) ^Δ^	0.274
	Uranues [15]	NR			29 (18.2)	30 (19.3)	0.910	24 (15.0)	24 (15.5)	0.489	1 (0.625) *	0 (0) *	NR	12 (5–71)	13 (4–142)	0.454
Systemic corticoids	Antila [14]	7 (41.18)	7 (50)	NR	2 (12)	3 (21)	0.636	1 (6%)	3 (21)	0.304	0 (0)	0 (0)	0.999	8 (2–23)	7 (6–38)	0.652
	Tarvainen [8]	20 (67)	11 (37)	***0.024***	NR			6 (20)	0 (0)	***0.024***	0 (0) *	1 (3) *	NR	7 (5–10.5)	7 (5–8)	0.064
Analogues of somatostatin	Gaujoux [26]	NR			NR			NR			1 (1.2)	0 (0)	0.99	NR		
Anastomosis of the pancreatic stump	Antila [36]	3 (60)	1 (12.5)	**<*0.05***	NR			NR			0 (0) *	0 (0) *	NR	10 (7–15)	7 (6–9)	NR
	Kawai [28]	24 (38.7)	23 (37.7)	0.332	11 (17.7)	7 (11.4)	0.326	7 (11.3)	8 (13.1)	0.757	0 (0)	0 (0)	0.999	16 (8–130)	16 (7–98)	0.727
	Uemura [19]	15 (41.67)	21 (56.76)	0.244	8 (22.85)	22 (61.11)	**<*0.001***	3 (8.57)	1 (2.77)	0.434	1 (2.77)	0 (0)	1	20.1 ± 15.7 ^+^	16.9 ± 8.9 ^+^	0.285
Routine use of drains	Van Bodegraven [23]	NR			NR			29 (20)	21 (16)	***0.0045***	0 (0)	3 (2)	0.12	6 (5–8)	6 (4–7)	***0.026***
	Van Buren [22]	NR			15 (9)	38 (22)	***0.0004***	51 (29)	44 (26)	0.477	0 (0)	2 (1.17)	0.244	5 (4–6) ^Δ^	5 (4–7) ^Δ^	***0.022***
Closure of the stump	Hamilton [38]	21 (45.65)	26 (46.43	***0.001***	NR			NR			NR			8.37 ± 6.8 ^+^	7.28 ± 4.6 ^+^	0.35
	Kondo [18]	30 (49.1)	36 (61.2)	0.15	6 (9.8)	8 (13.5)	0.52	10 (16.3)	11 (18.6)	0.74	0 (0) *	0 (0) *	0.99	19 (14–30)	20 (13–28)	0.78
	Wennerblom [24]	24 (42.86)	29 (58)	0.120	11 (20)	11 (22)	0.765	2 (4)	4 (8)	0.562	0 (0)	0 (0)	NR	8 (2–35)	9 (2–114)	0.541
	Merdrignac [12]	83 (83)	67 (67.7)	***0.01***	26 (26)	20 (20.2)	0.51	15 (15)	9 (9.1)	0.2	0 (0)	1 (1.01)	NR	8 (6–11)	8 (7–11)	0.85
	Shubert [3]	15 (46.9)	22 (62.9)	0.19	NR			10 (31.3)	9 (25.7)	0.62	0 (0) *	0 (0) *	NR	5.94 (3–13)	5 (3–15)	0.31
	Landoni [25]	35 (48)	30 (41)	0.552	23 (32)	10 (14)	***0.009***	13 (18)	6 (8)	0.074	1 (1)	0 (0)	0.50	8 (6–12)	8 (6–13)	0.880
	Diener [42]	63 (35.59)	64 (36.57)	0.84	34 (19.21)	34 (19.42)	0.95	NR			6 (3.39)	6 (3.42)	0.98	15.1 ±13.5 ^+^	15.7 ± 14.7 ^+^	0.86
	Sumiyoshi [41]	38 (43.2)	36 (43.4)	0.98	NR			6 (6.8)	4 (4.8)	0.57	0 (0) *	0 (0) *	NR	12 (10–20) ^Δ^	13 (10–20) ^Δ^	0.99
Transpapillary stent	Frozanpor [34]	13 (69.2)	10 (27)	0.122	10 (38.5)	4 (14.8)	0.065	11 (42.3)	5 (18.5)	0.065	0 (0) *	0 (0) *	NR	19.4 ± 14.4 ^+^	13.4 ± 6.4 ^+^	0.071

NR: Not reported; * 30-day mortality; ^Δ^ Value reported in median (interquartile range); ^+^ Value reported in mean (standard deviation). The figures in bold and italics are to highlight significant values.

## Data Availability

Data is available upon request to the corresponding author.

## Supplementary Materials

The following supporting information can be downloaded at: https://www.mdpi.com/article/10.3390/jcm15041433/s1, Table S1: Quality assessment of the studies using the ROB 2 Tool; Table S2: GRADE assessment of certainty of evidence across mitigation strategy categories. Table S3: PRISMA checklist.

## References

[1] Loos M., Mack C.E., Xu A.T.L., Hassenpflug M., Hinz U., Mehrabi A., Berchtold C., Schneider M., Al-Saeedi M., Roth S. (2023). Distal Pancreatectomy: Extent of resection determines surgical risk categories. Ann. Surg..

[2] Karabicak I., Satoi S., Yanagimoto H., Yamamoto T., Yamaki S., Kosaka H., Hirooka S., Kotsuka M., Michiura T., Inoue K. (2017). Comparison of surgical outcomes of three different stump closure techniques during distal pancreatectomy. Pancreatology.

[3] Shubert C.R., Ferrone C.R., Fernandez-del Castillo C., Kendrick M.L., Farnell M.B., Smoot R.L., Truty M.J., Que F.G. (2016). A multicenter randomized controlled trial comparing pancreatic leaks after TissueLink versus SEAMGUARD after distal pancreatectomy (PLATS) NCT01051856. J. Surg. Res..

[4] Chikhladze S., Makowiec F., Küsters S., Riediger H., Sick O., Fichtner-Feigl S., Hopt U.T., Wittel U.A. (2020). The rate of postoperative pancreatic fistula after distal pancreatectomy is independent of the pancreatic stump closure technique—A retrospective analysis of 284 cases. Asian J. Surg..

[5] Bassi C., Dervenis C., Butturini G., Fingerhut A., Yeo C., Izbicki J., Neoptolemos J., Sarr M., Traverso W., Buchler M. (2005). Postoperative pancreatic fistula: An international study group (ISGPF) definition. Surgery.

[6] Bassi C., Marchegiani G., Dervenis C., Sarr M., Abu Hilal M., Adham M., Allen P., Andersson R., Asbun H.J., Besselink M.G. (2017). The 2016 update of the International Study Group (ISGPS) definition and grading of postoperative pancreatic fistula: 11 Years After. Surgery.

[7] Park J.S., Lee D., Jang J., Han Y., Yoon D.S., Kim J.K., Han H.S., Yoon Y.S., Hwang D.W., Kang C.M. (2016). Use of TachoSil^®^ patches to prevent pancreatic leaks after distal pancreatectomy: A prospective, multicenter, randomized controlled study. J. Hepatobiliary Pancreat. Sci..

[8] Tarvainen T., Sirén J., Kokkola A., Sallinen V. (2020). Effect of Hydrocortisone vs. Pasireotide on Pancreatic Surgery Complications in Patients with High Risk of Pancreatic Fistula: A Randomized Clinical Trial. JAMA Surg..

[9] Hackert T., Klaiber U., Hinz U., Kehayova T., Probst P., Knebel P., Diener M.K., Schneider L., Scheneider L., Strobel O. (2017). Sphincter of Oddi botulinum toxin injection to prevent pancreatic fistula after distal pancreatectomy. Surgery.

[10] Andrianello S., Marchegiani G., Malleo G., Masini G., Balduzzi A., Paiella S., Esposito A., Landoni L., Casetti L., Tuveri M. (2020). Pancreaticojejunostomy with Externalized Stent vs. Pancreaticogastrostomy with Externalized Stent for Patients with High-Risk Pancreatic Anastomosis: A Single-Center, Phase 3, Randomized Clinical Trial. JAMA Surg..

[11] Mungroop T.H., Van Der Heijde N.V., Busch O.R., De Hingh I.H., Scheepers J.J., Dijkgraaf M.G., Koerkamp B.G., Besselink M.G., Van Eijjck C.H. (2021). Randomized clinical trial and meta-analysis of the impact of a fibrin sealant patch on pancreatic fistula after distal pancreatectomy: CPR trial. BJS Open.

[12] Merdrignac A., Garnier J., Dokmak S., Regenet N., Lesurtel M., Mabrut J.Y., Sa Cunha A., Fuks D., Bergeat D., Robin F. (2022). Effect of the use of reinforced stapling on the occurrence of pancreatic fistula after distal pancreatectomy results of the REPLAY (Reinforcement of the Pancreas in Distal Pancreatectomy) multicenter randomized clinical trial. Ann. Surg..

[13] Park Y., Ko J.H., Kang D.R., Lee J.H., Hwang D.W., Lee J.H., Hwang D.W., Lee W., Kwon J., Park S.N. (2020). Effect of flowable thrombin-containing collagen-based hemostatic matrix for preventing pancreatic fistula after pancreatectomy: A randomized clinical trial. J. Clin. Med..

[14] Antila A., Siiki A., Sand J., Laukkarinen J. (2019). Perioperative hydrocortisone treatment reduces postoperative pancreatic fistula rate after open distal pancreatectomy. A randomized placebo-controlled trial. Pancreatology.

[15] Uranues S., Fingerhut A., Belyaev O., Zerbi A., Boggi U., Hoffmann M.W., Reim D., Esposito A., Primavesi F., Kornprat P. (2021). Clinical Impact of Stump Closure Reinforced With Hemopatch on the Prevention of Clinically Relevant Pancreatic Fistula After Distal Pancreatectomy: A Multicenter Randomized Trial. Ann. Surg..

[16] Hassenpflug M., Hinz U., Strobel O., Volpert J., Knebel P., Diener M.K., Doerr-Harim C., Werner J., Hackert T., Bûchler M.W. (2016). Teres ligament patch reduces relevant morbidity after distal pancreatectomy (the DISCOVER Randomized Controlled Trial). Ann. Surg..

[17] Ergenc M., Karpuz S., Ergenc M., Yegen C. (2021). Enhanced recovery after pancreatic surgery: A prospective randomized controlled clinical trial. J. Surg. Oncol..

[18] Kondo N., Uemura K., Nakagawa N., Okada K., Kuroda S., Sudo T., Hadano N., Matstukawa H., Satoh D., Sasaki M. (2019). A Multicenter, Randomized, Controlled Trial Comparing Reinforced Staplers with Bare Staplers During Distal Pancreatectomy (HiSCO-07 Trial). Ann. Surg. Oncol..

[19] Uemura K., Satoi S., Motoi F., Kwon M., Unno M., Murakami Y. (2017). Randomized clinical trial of duct-to-mucosa pancreaticogastrostomy versus handsewn closure after distal pancreatectomy. Br. J. Surg..

[20] Čečka F., Jon B., Skalický P., Čermáková E., Neoral Č., Loveček M. (2018). Results of a randomized controlled trial comparing closed-suction drains versus passive gravity drains after pancreatic resection. Surgery.

[21] Baba H., Oba A., Tanaka K., Miura T., Ban D., Edanami M., Ishikawa Y., Ohgi K., Tanaka H., Shintakuya R. (2024). The efficacy of wrapping with polyglycolic acid mesh and fibrin glue in preventing clinically relevant pancreatic fistula after minimally invasive distal pancreatectomy (WRAP Study): Study protocol for a multicenter randomized controlled trial in Japan. BMC Surg..

[22] Van Buren G., Bloomston M., Schmidt C.R., Behrman S.W., Zyromski N.J., Ball C.G., Morgan K.A., Hugher S.J., Karanicolas P.J., Allendorf J.D. (2017). A Prospective Randomized Multicenter Trial of Distal Pancreatectomy with and Without Routine Intraperitoneal Drainage. Ann. Surg..

[23] van Bodegraven E.A., Balduzzi A., van Ramshorst T.M.E., Malleo G., Vissers F.L., van Hilst J., Festen S., Abu Hilal M., Asbun H.J., Michiels N. (2024). Prophylactic abdominal drainage after distal pancreatectomy (PANDORINA): An international, multicentre, open-label, randomised controlled, non-inferiority trial. Lancet Gastroenterol. Hepatol..

[24] Wennerblom J., Ateeb Z., Jönsson C., Björnsson B., Tingstedt B., Williamsson C., Sandstròm P., Ansorge C., Blomberg J., Del Chiaro M. (2021). Reinforced versus standard stapler transection on postoperative pancreatic fistula in distal pancreatectomy: Multicentre randomized clinical trial. Br. J. Surg..

[25] Landoni L., De Pastena M., Fontana M., Malleo G., Esposito A., Casetti L., Marchegiani G., Tuveri M., Paiella S., Pea A. (2022). A randomized controlled trial of stapled versus ultrasonic transection in distal pancreatectomy. Surg. Endosc..

[26] Gaujoux S., Regimbeau J.M., Piessen G., Truant S., Foissac F., Barbier L., Buc E., Adham M., Fuks D., Deguelte S. (2024). Somatostatin Versus Octreotide for Prevention of Postoperative Pancreatic Fistula: The PREFIPS Randomized Clinical Trial: A FRENCH 007-ACHBT Study. Ann. Surg..

[27] Jang J.Y., Shin Y.C., Han Y., Park J.S., Han H.S., Hwang H.K., Yoon D.S., Kim J.K., Yoon Y.S., Hwang D.W. (2017). Effect of polyglycolic acid mesh for prevention of pancreatic fistula following distal pancreatectomy: A randomized clinical trial. JAMA Surg..

[28] Kawai M., Hirono S., Okada K.I., Sho M., Nakajima Y., Eguchi H., Nagano H., Ikoma H., Morimura R., Takeda Y. (2016). Randomized controlled trial of pancreaticojejunostomy versus stapler closure of the pancreatic stump during distal pancreatectomy to reduce pancreatic fistula. Ann. Surg..

[29] Page M.J., McKenzie J.E., Bossuyt P.M., Boutron I., Hoffmann T.C., Mulrow C.D., Shamseer L., Tetzlaff J.M., Akl E.A., Brennan S.E. (2021). The PRISMA 2020 statement: An updated guideline for reporting systematic reviews. BMJ.

[30] Dindo D., Demartines N., Clavien P.A. (2004). Classification of surgical complications: A new proposal with evaluation in a cohort of 6336 patients and results of a survey. Ann. Surg..

[31] Sterne J.A.C., Savović J., Page M.J., Elbers R.G., Blencowe N.S., Boutron I., Cates C.J., Cheng H.Y., Corbett M.S., Eldridge S.M. (2019). RoB 2: A revised tool for assessing risk of bias in randomised trials. BMJ.

[32] Schünemann H., Brożek J., Guyatt G., Oxman A. (2023). GRADE Handbook for Grading Quality of Evidence and Strength of Recommendations. https://gdt.gradepro.org/app/handbook/handbook.html.

[33] Guyatt G.H., Oxman A.D., Vist G.E., Kunz R., Falck-Ytter Y., Alonso-Coello P., Schûnemann H.J., GRADE Working Group (2008). GRADE: An emerging consensus on rating quality of evidence and strength of recommendations. BMJ.

[34] Frozanpor F., Lundell L., Segersvärd R., Arnelo U. (2012). The effect of prophylactic Transpapillary pancreatic stent insertion on clinically significant leak rate following distal pancreatectomy: Results of a prospective controlled clinical trial. Ann. Surg..

[35] Carter T.I., Fong Z.V., Hyslop T., Lavu H., Tan W.P., Hardacre J., Sauter P.K., Kennedy E.P., Yeo C.J., Rosato E.L. (2013). A Dual-Institution Randomized Controlled Trial of Remnant Closure after Distal Pancreatectomy: Does the Addition of a Falciform Patch and Fibrin Glue Improve Outcomes?. J. Gastrointest. Surg..

[36] Antila A., Sand J., Nordback I., Räty S., Laukkarinen J. (2014). Is Roux-Y binding pancreaticojejunal anastomosis feasible for patients undergoing left pancreatectomy? Results from a prospective randomized trial. Biomed Res. Int..

[37] Sa Cunha A., Carrere N., Meunier B., Fabre J.M., Sauvanet A., Pessaux P., Ortega-Deballon P., Fingerhut A., Lacaine F., FRENCH (2015). Stump closure reinforcement with absorbable fibrin collagen sealant sponge (TachoSil) does not prevent pancreatic fistula after distal pancreatectomy: The FIABLE multicenter controlled randomized study. Am. J. Surg..

[38] Hamilton N.A., Porembka M.R., Johnston F.M., Gao F., Strasberg S.M., Linehan D.C., Hawkins W.G. (2012). Mesh reinforcement of pancreatic transection decreases incidence of pancreatic occlusion failure for left pancreatectomy: A single-blinded, randomized controlled trial. Ann. Surg..

[39] Montorsi M., Zerbi A., Bassi C., Capussotti L., Coppola R., Sacchi M., Italian Tachosil Study Group (2012). Efficacy of an absorbable fibrin sealant patch (TachoSil) after distal pancreatectomy: A multicenter, randomized, controlled trial. Ann. Surg..

[40] Oláh A., Issekutz Á., Belágyi T., Hajdú N., Romics L. (2009). Randomized clinical trial of techniques for closure of the pancreatic remnant following distal pancreatectomy. Br. J. Surg..

[41] Sumiyoshi T., Uemura K., Seo S., Fujii T., Satoi S., Miwa T., Fukasawa M., Yamaki S., Oshita A., Abe T. (2025). Impact of pre-compression versus non-compression before parenchyma transection in left-sided pancreatic resection on the rate of clinically relevant pancreatic fistula: Multicentre randomized clinical trial. Br. J. Surg..

[42] Diener M.K., Seiler C.M., Rossion I., Kleeff J., Glanemann M., Butturini G., Tomazic A., Bruns C.J., Busch O.R.C., Farkas S. (2011). Efficacy of stapler versus hand-sewn closure after distal pancreatectomy (DISPACT): A randomised, controlled multicentre trial. Lancet.

[43] Sallinen V., Tarvainen T. (2020). The Hydrocortisone vs. Pasireotide in Reducing Pancreatic Surgery Complications Noninferiority Trial—Reply. JAMA Surg..

[44] Aranha G.V., Aaron J.M., Shoup M., Pickleman J. (2006). Current management of pancreatic fistula after pancreaticoduodenectomy. Surgery.

[45] Xia N., Li J., Huang X., Tian B., Xiong J. (2023). Reinforced stapling does not reduce postoperative pancreatic fistula in distal pancreatectomy: A systematic review and meta-analysis. Updates Surg..

[46] Kollár D., Huszár T., Pohárnok Z., Cselovszky É., Oláh A. (2016). A Review of Techniques for Closure of the Pancreatic Remnant following Distal Pancreatectomy. Dig. Surg..

[47] Lai M., Zhou S., He S., Cheng Y., Cheng N., Deng Y., Ding X. (2023). Fibrin sealants for the prevention of postoperative pancreatic fistula following pancreatic surgery. Cochrane Database Syst. Rev..

[48] Hüttner F.J., Mihaljevic A.L., Hackert T., Ulrich A., Büchler M.W., Diener M.K. (2016). Effectiveness of Tachosil^®^ in the prevention of postoperative pancreatic fistula after distal pancreatectomy: A systematic review and meta-analysis. Langenbecks Arch. Surg..

[49] Park S.M., Kang D.R., Lee J.H., Jeong Y.H., Shin D.A., Yi S., Ha Y., Kim K.N. (2021). Efficacy and Safety of a Thrombin-Containing Collagen-Based Hemostatic Agent in Spinal Surgery: A Randomized Clinical Trial. World Neurosurg..

[50] Murata Y., Komatsubara H., Noguchi D., Ito T., Hayasaki A., Iizawa Y., Fujii T., Tanemura A., Kuriyama N., Kishiwada M. (2025). Effect of Transpancreatic Mattress Suture With Polyglycolic Acid Sheet in Pancreatic Stump Closure for the Prevention of Postoperative Pancreatic Fistula in Robotic Distal Pancreatectomy. Surg. Laparosc. Endosc. Percutaneous Tech..

[51] Zhang W., Wei Z., Che X. (2020). Effect of polyglycolic acid mesh for prevention of pancreatic fistula after pancreatectomy. Medicine.

[52] De Pastena M., van Bodegraven E.A., Mungroop T.H., Vissers F.L., Jones L.R., Marchegiani G., Balduzzi A., Klompmaker S., Paiella S., Rad S.T. (2023). Distal Pancreatectomy Fistula Risk Score (D-FRS). Ann. Surg..

[53] van Bodegraven E.A., den Haring F.E.T., Pollemans B., Monselis D., De Pastena M., van Eijck C., Daams F., Hingh I., Luyer M., Stommel M.W.J. (2023). Nationwide validation of the distal fistula risk score (D-FRS). Langenbecks Arch. Surg..

[54] Pecorelli N., Ricci C., Esposito A., Capretti G., Partelli S., Butturini G., Boggi U., Cucchetti A., Zerbi A., Salvia R. (2024). Italian survey about intraperitoneal drain use in distal pancreatectomy. Updates Surg..

[55] Hajibandeh S., Mostafa O.E.S., Akula Y., Ghassemi N., Hajibandeh S., Bhatt A., Durkin D., Athwal T.S., Laing R.W. (2024). Meta-analysis of routine abdominal drainage versus no drainage following distal pancreatectomy: Does the best available evidence overcome “HPB surgeon’s paranoia”?. Pancreatology.

[56] Kuan L.L., Dennison A.R., Garcea G. (2021). Outcomes of peri-operative glucocorticosteroid use in major pancreatic resections: A systematic review. HPB.

[57] Czarnecka Z., Verhoeff K., Bigam D., Dajani K., Shapiro J., Anderson B. (2025). Impact of soft pancreas on pancreaticoduodenectomy outcomes and the development of the preoperative soft pancreas risk score. Ann. Hepatobiliary Pancreat. Surg..

[58] Kawaida H., Kono H., Amemiya H., Hosomura N., Saito R., Takahashi K., Yamamoto A., Watanabe M., Furuya S., Shimizu H. (2019). Use of a Reinforced Triple-row Stapler Following Distal Pancreatectomy Reduces the Incidence of Postoperative Pancreatic Fistula in Patients With a High BMI. Anticancer Res..

[59] Matsumoto I., Kamei K., Satoi S., Murase T., Matsumoto M., Kawaguchi K., Yoshida Y., Dongha L., Takebe A., Nakai T. (2022). Efficacy of the slow firing method using a reinforced triple-row stapler for preventing postoperative pancreatic fistula during laparoscopic distal pancreatectomy. Surg. Today.

[60] Kang M.K., Kim H., Byun Y., Han Y., Choi Y.J., Kang J.S., Kwon W., Han I.W., Shin S.H., Choi D.W. (2021). Optimal stapler cartridge selection to reduce post-operative pancreatic fistula according to the pancreatic characteristics in stapler closure distal pancreatectomy. HPB.

