# Supplementary material for: Evidence-Based Strategies for Mitigating Pancreatic Fistula After Distal Pancreatectomy: A Systematic Review of Randomized Clinical Trials
Source: J Clin Med. 2026 Feb 12;15(4):1433. doi: 10.3390/jcm15041433 (PMC12942036; doi:10.3390/jcm15041433)
Supplement: Supplementary file 1 [file jcm-15-01433-s001.zip › jcm-4046752-supplementary.pdf]

Table S1. Quality assessment of the studies using the ROB 2 Tool

Study	*Type of analysis*	*Randomisation process*	*Deviation from intended interventions*	*Missing outcome data*	*Measurement of the outcome*	*Selection of the reported result*	***Overall***
Olah, 2009 [1]	ITT	+	!	+	+	+	+
Carter, 2013[2]	ITT	+	+	+	+	+	+
Hassenpflug, 2016 [3]	ITT	+	!	+	+	+	!
Jang, 2017 [4]	ITT	+	!	+	+	+	!
Montorsi, 2012 [5]	ITT	!	!	+	+	+	!
Sa Cunha, 2015 [6]	ITT	+	+	+	+	+	+
Park, 2016 [7]	PP	!	+	+	+	+	!
Mungroop, 2021 [8]	PP	!	+	+	+	+	!
Park, 2020 [9]	ITT	+	+	+	+	+	+
Uranues, 2021 [10]	PP	!	+	+	+	+	!
Antila, 2019 [11]	PP	+	!	+	+	+	+
Tarvainen, 2020 [12]	PP	+	+	+	+	+	+
Gaujoux, 2024 [13]	ITT	+	!	+	!	!	!
Antila 2014 [14]	ITT	!	+	+	+	!	!
Kawai, 2016 [15]	PP	!	!	+	+	+	!
Uemura, 2017 [16]	PP	!	+	+	+	+	!
Van Bodegraven, 2024 [17]	ITT	+	+	+	+	+	+
Van Buren, 2017 [18]	PP	+	+	+	+	+	+
Hamilton, 2012 [19]	ITT	!	!	+	+	+	!
Kondo, 2019 [20]	ITT	+	+	+	+	+	+

Wennerblom, 2021 [21]	ITT	+	+	+	+	+	+
Merdrignac, 2022 [22]	ITT	+	+	+	+	+	+
Shubert, 2016 [23]	ITT	!	+	+	+	!	!
Landoni, 2022 [24]	PP	+	+	+	+	+	+
Diener, 2011 [25]	ITT	+	+	+	+	+	+
Sumiyoshi, 2025 [26]	ITT	+	+	+	+	+	+
Frozanpor, 2012 [27]	PP	+	+	+	+	+	+

+ Low risk, ! Some concerns, − High risk

ITT: Intention-to-treat analysis, PP: Per-protocol analysis

Table S2. GRADE assessment of certainty of evidence across mitigation strategy categories

Strategy category	n of included studies	Effect on CR-POPF (success)	Risk of Bias *	Inconsistency	Indirectness	Imprecision	Publication bias	Certainty (GRADE)	Comments
Tissue coverage	4	1/4	2 low risk, 2 some concerns	Yes	Yes – Population and intervention heterogeneity.	No	Possible-few studies	**Low**	Heterogeneity in patient selection and techniques; only one trial showed benefit
Sealants and glues	6	1/6	2 low risk, 4 some concerns	Yes	Yes – intervention heterogeneity	Yes – Only positive result from a small trial; effect estimates from larger studies inconclusive.	No	**Low**	Heterogeneous interventions; effect not consistent; sample size acceptable overall
Systemic corticoids	2	1/2	Low risk	Yes	Yes - heterogeneity in comparators	Yes – Total sample size <100	No	**Low**	Very limited data; conflicting results; comparator and size limit confidence in conclusions
Analogues of somatostatin	2	0/2	1 Low risk, 1 some concerns	No	Yes – Both trials used active comparators	Yes – Total sample size moderate	No	**Low**	Active comparators, few studies, limiting the ability to isolate their true effect
Anastomosis of the pancreatic stump	3	$0/3^{+}$	Some concerns	Yes	Yes – Heterogeneity in surgical approach	Yes – Small total sample size and few events	No	**Low**	Results are inconsistent and affected by surgical variability; clinical

									benefit remains unclear
Routinary use of drains	2	1/2	Low risk	Yes	No	No – Sample size adequate	No	**Moderate**	Large, well-conducted trials with diverging results; evidence supports selective over routine drainage
Closure of the stump	8	1/8	6 Low risk /2 Some concerns	Yes	Yes – intervention heterogeneity	Yes –Overall effect remains uncertain without pooled analysis.	No	**Very low**	Contrasting results, not enough evidence to conclude formally
Transpapillary stent	1	0/1	Low risk	Not applicable – single study	No	Yes- wide confidence interval	Yes- single small study	**Very low**	Evidence from a single small RCT with a wide confidence interval; effect remains highly uncertain.

GRADE: Grading of Recommendations Assessment, Development and Evaluation
CR-POPF: Clinically relevant postoperative pancreatic fistula
* Risk of bias assessed with the ROB 2 Tool
$^{+}$ Only one study was statistically significant in favor to handsewn technique vs pancreticojejunal anastomosis
